# Multi-Parametric Exploration of a Selection of Piezoceramic Materials for Bone Graft Substitute Applications

**DOI:** 10.3390/ma16030901

**Published:** 2023-01-17

**Authors:** Liviu Nedelcu, José M. F. Ferreira, Adrian-Claudiu Popa, Luminița Amarande, Bo Nan, Liliana-Marinela Bălescu, Cezar Dragoș Geambașu, Marius-Cristian Cioangher, Lucia Leonat, Mihai Grigoroscuță, Daniel Cristea, Hermine Stroescu, Robert Cătălin Ciocoiu, George E. Stan

**Affiliations:** 1 National Institute of Materials Physics, 077125 Magurele, Romania; 2 Department of Materials and Ceramic Engineering, CICECO—Aveiro Materials Institute, University of Aveiro, 3810-193 Aveiro, Portugal; 3 Department of Materials Science, Faculty of Materials Science and Engineering, Transilvania University of Brasov, 500068 Brasov, Romania; 4 “Ilie Murgulescu” Institute of Physical Chemistry of the Romanian Academy, 060021 Bucharest, Romania; 5 Department of Metallic Materials Science, Physical Metallurgy, University Politehnica of Bucharest, 060042 Bucharest, Romania

**Keywords:** piezoceramics, physico-chemical characterization, in vitro testing, robocasting, macro-porous scaffolds, bone graft substitutes

## Abstract

This work was devoted to the first multi-parametric unitary comparative analysis of a selection of sintered piezoceramic materials synthesised by solid-state reactions, aiming to delineate the most promising biocompatible piezoelectric material, to be further implemented into macro-porous ceramic scaffolds fabricated by 3D printing technologies. The piezoceramics under scrutiny were: KNbO_3_, LiNbO_3_, LiTaO_3_, BaTiO_3_, Zr-doped BaTiO_3_, and the (Ba_0.85_Ca_0.15_)(Ti_0.9_Zr_0.1_)O_3_ solid solution (BCTZ). The XRD analysis revealed the high crystallinity of all sintered ceramics, while the best densification was achieved for the BaTiO_3_-based materials via conventional sintering. Conjunctively, BCTZ yielded the best combination of functional properties—piezoelectric response (in terms of longitudinal piezoelectric constant and planar electromechanical coupling factor) and mechanical and in vitro osteoblast cell compatibility. The selected piezoceramic was further used as a base material for the robocasting fabrication of 3D macro-porous scaffolds (porosity of ~50%), which yielded a promising compressive strength of ~20 MPa (higher than that of trabecular bone), excellent cell colonization capability, and noteworthy cytocompatibility in osteoblast cell cultures, analogous to the biological control. Thereby, good prospects for the possible development of a new generation of synthetic bone graft substitutes endowed with the piezoelectric effect as a stimulus for the enhancement of osteogenic capacity were settled.

## 1. Introduction

Bone is the second-most transplanted tissue in the human body after blood [1,2]. The self-healing ability of bone is well documented. However, this is limited to defects with a maximum critical size of ~10 mm, beyond which the natural healing process is impeded [3,4]. Such large-sized bone defects stem from bone deficiency or substantial skeletal loss, and have multiple causes: e.g., age, severe trauma as a result of accidents, chronic bone disorders, infections, bone tumor resections, or congenital conditions [2,3]. Lately, the frequency of such medical afflictions increased as a consequence of a series of independent or conjugated factors: (i) life expectancy is increasing yearly by 2–3% [4,5]; (ii) global population grows at an annual rate of 1.1% (i.e., ~83 million); (iii) more people are inclined to adopt a more active lifestyle (with the skeletal system becoming more strained); (iv) societal awareness of progresses made by nowadays medicine; and (v) accessibility to high-quality medical services is increasing worldwide. Inevitably, bone grafting evolved in the last decade into a distinct biomedical segment [1,2,3,4,6,7,8,9,10]. Presently, it is recognised that more than 20 million people suffer from bone diseases [3], and around 2 million bone graft-based medical interventions are carried out every year in the world [1,3,11]. Apart from improving the quality of life of patients and the societal benefits emerging from it, a significant economic impact is also expected. In fact, reputed financial research agencies forecast that the bone grafts sector, evaluated in 2021 at USD ~2.9 billion, is predicted to record an increased compound annual growth rate of ~6.2–7.5% from 2022 to 2030 [12,13].

Bone grafts can be classified into four large families: autografts (currently considered the gold standard), allografts, xenografts, and synthetic bone graft substitutes (currently on the rise) [11,14]. The advantages of each class of bone grafts were recently reviewed in a series of works [15,16,17,18,19], and thus, here, only a brief synopsis will be given for the reader’s benefit. Autografts, surgically harvested from a healthy bone site (e.g., iliac crest, tibia, calvarium) of the patient and transplanted to the affected site, are known for their excellent histocompatibility, osteogenicity, and low risk of immunological rejection; however, their availability is obviously quite limited, while the risk of donor site morbidity cannot be easily dismissed, and failure for specific bone sites has been recorded [11,14]. Allografts (harvested from living human donors or cadavers) and xenografts (collected from animal sources, mainly bovine and swine) are mainly marred by the risk of disease transmission and immune rejection [11,14,20]. To limit such biological hazards, allografts (in short supply) and xenografts (in higher abundance) need to be decellularized and devitalised, which alters their primary mechanical properties [14]. Nevertheless, precision shaping of autografts, allografts, or xenografts for particular and irregular patient osseous defects is both a time-consuming and highly complicated task (furthermore in the surgery room).

The fourth class of bone grafting solutions is constituted by the synthetic bone graft substitutes (SBGS), which imply the fabrication of porous, yet mechanically resistant constructs, of metallic, ceramic, polymeric, or composite origin [3,14,20,21,22]. So far, most SBGS constructs are based on calcium phosphates (CaPs)—hydroxyapatite [HA, Ca_10_(PO_4_)_6_(OH)_2_] and beta-tricalcium phosphate [β-TCP, β-Ca_3_(PO_4_)_2_] or a blend of them [11,22,23,24,25]. In fact, a series of commercial products are already available: e.g., MBCP^®^, Triosite^®^, Ceraform^®^, Bio-Oss^®^, to name just a few. Although undeniable progress has been made in this field, ideal SBGS constructs are still far from reality. No standard design for SBGS exists yet. SBGSs should possess a 3D hierarchical porous architecture and meet definite requirements: suitable biological features (i.e., permeability to allow cell migration, nutrient flow, and oxygen supply; cell invasion, adhesion, viability, and proliferation; controlled degradability; angiogenesis; osteogenesis) coupled with dependable mechanical properties. In fact, currently, the vast majority of SBGSs are envisioned for trabecular bone reconstructions, since in such a case the mechanical performance requirements are less demanding, and thereby easier to fulfil by the macro-porous constructs made of conventional bioactive CaP-based ceramics [14,20,26]. Furthermore, the cortical bone is known to have a lower regeneration speed than the trabecular one [27], and thereby, the SBGS constructs would require not only superior mechanical properties, but also lower degradation rates and supplemental osteogenic triggers. 

To this effect, high-temperature sintered lead-free piezoelectric ceramics [28,29,30,31] could definitely be viewed as promising candidates. However, even if the piezoelectricity effect discovered in bone [32] offered a rationale for the accelerated bone remodelling under mechanical stress (effecting in the electric charge accumulation on the surface of bone crystallites) [33,34], the exploration of piezoelectric ceramics capable to boost bone restoration and healing and thus, be integrated into SBGS designs, still remains a rarely approached niche of research. This study was also motivated by the fact that, even if the basic cytocompatibility [35,36,37,38,39,40,41,42] and biomineralization ability [36,40] of such piezoelectric compounds have been suggested (although only sporadically explored), no intercomparison unitary study (comparing and testing at least two such piezoceramics with equivalent investigation techniques) has yet been performed to the best of our knowledge. Consequently, this work was devoted to the multi-parametrical analysis (markedly, morphological, structural, electrical, mechanical, and in vitro biological investigations) of a series of noteworthy lead-free sintered piezoceramics (i.e., KNbO_3_, LiNbO_3_, LiTaO_3_, BaTiO_3_, Zr-doped BaTiO_3_ and the solid solution of the binary system BaZr_0.2_Ti_0.8_O_3_–Ba_0.7_Ca_0.3_TiO_3_—BCTZ), aiming to delineate the most suitable piezoelectric ceramic candidate material for bone defect reconstruction applications. Furthermore, in this respect, pilot studies of 3D printing (by robocasting) of SBGS constructs (from the thereby selected material) and their mechanical and cytocompatibility responses were preliminarily evaluated and discussed.

## 2. Materials and Methods

### 2.1. Preparation of Piezoceramic Materials

A series of noteworthy lead-free sintered piezoceramics (i.e., KNbO_3_, LiNbO_3_, LiTaO_3_, BaTiO_3_, Zr-doped BaTiO_3_ and a solid solution of the binary system BaZr_0.2_Ti_0.8_O_3_–Ba_0.7_Ca_0.3_TiO_3_—BCTZ) have been selected and scrutinised in this work. All these materials were synthesised by the conventional solid-state reaction method, mixing (with a high energy agate ball mill) stoichiometrically adequate oxide or carbonate (reagent grade, ≥99.5%) powders (Sigma-Aldrich, St. Louis, MO, USA) as indicated below:

- BaTiO_3_ (BT)—reagents: BaCO_3_ and TiO_2_;
- Zr-doped (2 mol%) BT (Zr:BT)—reagents: BaCO_3_, TiO_2_ and ZrO_2_;
- (Ba_0.85_Ca_0.15_)(Ti_0.9_Zr_0.1_)O_3_ solid solution (BCTZ50)—reagents: BaCO_3_, CaCO_3_, TiO_2_ and ZrO_2_;
- KNbO_3_ (KNO)—reagents: K_2_CO_3_ and Nb_2_O_5_;
- LiNbO_3_ (LNO)—reagents: Li_2_CO_3_ and Nb_2_O_5_;
- LiTaO_3_ (LTO)—reagents: Li_2_CO_3_ and Ta_2_O_5_.

The calcination conditions for each compound are given in Table 1. The final powder blends were sieved and mixed with polyvinyl alcohol (as a binding agent), and further uniaxially pressed in cylindrical moulds (13 mm diameter) to achieve pellets with green density >60%. The pressed pellets were further sintered in air at different temperatures and durations, aiming to retain the targeted single-phase ceramic structure, while obtaining a high densification. The performed experiments indicated that KNO and LTO ceramics were difficult to densify by conventional (hereinafter marked as “conv.”) sintering processes (even in the presence of MnO and Fe_2_O_3_ dopants [43]—results not shown). In these cases (i.e., KTO and LTO), the spark plasma sintering (SPS) technique was employed for enhancing densification. SPS was performed on an FCT GmbH System—HP D 5 (Effelder-Rauenstein, Germany), using 5 g of calcined KNO or LTO powders and a graphite mould with a diameter of 20 mm. The carbon that diffused from graphite into the samples was removed by thermally treating the samples in air, at 900 °C, for 10 h. Irrespective of the sintering procedure, KNO elicited a peculiar behaviour, being extremely hygroscopic and thus highly deliquescent (easily and readily absorbing ambient moisture and dissolving). Therefore, KNO cannot be considered a good candidate for the further integration in SBGS constructs, due to its expected fast disintegration in contact with physiological fluids, and was disregarded for the functional (electrical and biological) studies. The processing parameters that led to the fabrication of the highest-density disks, with single-phase composition, are presented in Table 1.

For the morphological (i.e., SEM analysis) and electrical (i.e., ferroelectric, piezoelectric and dielectric measurements) investigations, the sintered ceramic cylinders were plane-parallel sectioned by diamond wheel cutting, obtaining disks with a thickness of ~1 mm; their surface was further polished with SiC-abrasive papers down to grit 1000. Part of these disks were milled in form of fine powders to perform the XRD characterizations.

### 2.2. Fabrication of Piezoceramic Macro-Porous Scaffolds by Robocasting

The selected piezoelectric material, with a median (D_50_) particle size of ~1 µm, was further used to prepare a ceramic paste. A water-based ceramic paste, adequate for robocasting printing, could be prepared by the successive addition of a series of additives—Dispex^®^ AA4040 (BASF SE, Ludwigshafen, Germany), hydroxypropyl methylcellulose (HPMC, Methocel™ F4M (DuPont—ChemPoint, Bellevue, WA, USA), and polyethyleneimine (PEI, average molecular weight M_n_ ≈ 1800, Sigma-Aldrich, St. Louis, MO, USA) as dispersant, binder, and jellifying agents, respectively. Dispersant, HPMC and PEI quantities of ~1, 2 and 0.05 wt.%, respectively (relative to the powder mass), were needed to obtain a ceramic paste with a pseudoplastic (shear-thinning) behaviour, having a solid loading of ~41 vol.%. The homogenization of the suspension/paste after the addition of the dispersant, binder and jellifying agents was performed in a planetary mixer (Thinky ARE-250-CE, Thinky USA Inc., Laguna Hills, CA, USA) using speeds of 600, 1300 and 1500 rotations/min, respectively, and a dwell time of 5 min.

Cylindrical macro-porous scaffolds (with a diameter and height of 8 and ~5.8 mm, respectively), consisting of 26 consecutive layers deposited in a sequential 45 degrees-rotated printing pattern, were fabricated in ambient conditions with the help of a robocasting mechanical motor-driven system (3D Inks, LLC, Tulsa, OK, USA). Nordson EFD printing nozzles (Nordson Corporation, Westlake, OH, USA) with a diameter of 250 μm were employed. An edge-to-edge inter-filament distance of 200 μm was used. This design allows for a complex tortuosity of the macro-porous scaffold, mimicking the convoluted assembly of the bone trabeculae, and it was recently found to enable good cell colonization [25].

Subsequent to printing, the scaffolds were subjected to a four-step post-processing: (i) dry naturally overnight at room temperature (RT) for 24 h; (ii) removal of moisture at 200 °C in a forced air oven for 24 h; (iii) complete elimination of the organic additives at 800 °C/2 h (heating rate of 1 °C/min; natural cooling to RT in ~2 h); and (iv) sintering at 1500 °C/2.5 h (heating rate of 3 °C/min; natural cooling to RT in ~12 h).

### 2.3. Structural, Morphological, Electrical, and Mechanical Characterization

The crystalline structure of the piezoceramic specimens was investigated by X-ray diffraction (XRD) in Bragg–Brentano mode with the help of a Bruker D8 Advance diffractometer (Bruker AXS Advanced X-ray Solutions GmbH, Karlsruhe, Germany), equipped with a one-dimensional LynxEye**^®^**-type detector (Bruker AXS Advanced X-ray Solutions GmbH, Karlsruhe, Germany) and a tube with CuK_α_ radiation (λ = 1.5418 Å). The XRD diagrams were recorded over the 2θ range 15–65°, with a step of 0.02°, and a dwell time of 2 s/step. For an accurate determination of the full-width at half maximum (FWHM) of diffraction peaks, the instrumental broadening was inferred using a National Institute of Standards and Technology (NIST, Gaithersburg, MD, USA) standard reference material (SRM): corundum NIST-SRM 1976.

The density of the sintered ceramic disks was calculated by applying the principle of Archimedes with distilled water as the liquid medium. For this scope, a Sartorius Cubis**^®^** MSA224S analytical micro-balance (Sartorius AG, Göttingen, Germany) having a readability of 0.1 mg and equipped with a density determination kit (YDK01MS), was used.

The morphology analyses were performed using a field emission scanning electron microscopy (FE-SEM) Carl Zeiss Gemini 500 system (Carl Zeiss Company, Oberkochen, Germany). No conductive coating application was required. The FE-SEM analyses were done under high vacuum (~10^−4^ Pa), at working distances of 5–7 mm and acceleration voltages of 3–5 kV.

The dielectric properties of the ceramic pellets were evaluated by capacitance and dielectric loss measurements performed at 1 kHz, with an Agilent 4294A (Santa Clara, CA, USA) impedance analyser. The ferroelectric properties were assessed with a Premier II-Radiant Technologies ferrotester (Radiant Technologies, Albuquerque, NM, USA) by measuring the total electric polarization P with respect to the bipolar electric field E (with triangular time dependence—3 s). For these measurements, silver paste electrodes were deposited on both faces of the disks, and subsequently heat-treated at 200 °C/1 h, to ensure proper adhesion. The poling of the ceramics was performed at RT, at fields of 3.5–4 kV/mm, such as to avoid the increase of electrical conductivity with respect to temperature and to prevent the electrical breakdown of the samples. The piezoelectric properties were studied in the quasi-static and resonant regimes. For the quasi-static regime assessments, a PM300 PiezoMeter system (Piezotest Pte. Ltd., Singapore) was employed. A force with an amplitude of 0.25 N was applied cyclically at a low frequency (110 Hz), conditions that were similar to the static regime. The resonant regime was implemented with an impedance analyser (Agilent 4294A (Santa Clara, CA, USA)).

The mechanical properties of the sintered ceramics were evaluated by instrumented indentation tests using an NHT-2 CSM Instruments module (Anton Paar GmbH, Peseux, Switzerland), equipped with a Berkovich three-sided diamond indenter. The modulus of elasticity (E) and hardness (H) of piezoceramics were calculated from the load–displacement curves, employing the Oliver–Pharr method [44].

The compressive strength measurements of the 3D-printed piezoceramic scaffolds were carried out using a Lloyd Instruments LRXPlus (Lloyd Instruments Ltd., Bognor Regis, UK), with the tests running in displacement control at a speed of 0.5 mm/min.

### 2.4. In Vitro Biological Assays

Prior to the biological in vitro testing, all materials (disks and scaffolds) were sterilised by a dry heat procedure performed at 180 °C/1 h.

#### 2.4.1. Sintered Disks

The pH determinations were performed in the same culture medium used in the framework of the cytocompatibility studies (presented hereinafter), namely Dulbecco’s Modified Eagle’s Medium/Nutrient Mixture F-12 (Sigma-Aldrich, St. Louis, MO, USA) supplemented with 10% foetal bovine serum (DMEM/F12-FBS). A volume of 500 µL of DMEM/F12-FBS medium was added to a sample surface with a nominal area of 100 mm^2^. Since the sintered pellets had different final sizes, the volume was adapted accordingly. The tests were carried out for 36 h, under the correct biomimetic conditions—5% CO_2_, 37 °C, humidified ambient in a biology-dedicated New Brunswick Galaxy^®^ 48 R incubator (Eppendorf, Hamburg, Germany). Subsequent to testing, the culture medium was extracted. A part of the medium was used for the determination of the pH, with the help of a Horiba LAQUAtwin pH-33 pH meter (Horiba Company, Kyoto, Japan), while another part of it was reserved for the inductively coupled plasma mass spectrometry (ICP-MS) analyses. Prior to each set of measurements, the pH meter was calibrated using standard buffers with pH values of 7 and 10, followed by the rinsing of the pH sensor in deionised water and drying by argon gas purging.

The cytocompatibility assessment was performed using an hFOB 1.19 human osteoblast cell line (ATCC^®^ CRL-11372™, ATCC, Manassas, VA, USA), considered a relevant in vitro model for the targeted application, i.e., bone regeneration. The tests were carried out in accordance with ISO 10993-5/2009: “Biological evaluation of medical devices—Part 5: Tests for in vitro cytotoxicity”. The cell viability/proliferation were assessed by a CellTiter 96^®^ (3-(4,5-dimethyl thiazol-2-yl)5-(3-carboxymethoxyphenyl)-2-(4-sulfophenyl)-2H-tetrazolium) (MTS) assay (Promega Corporation, Madison, WI, USA). The cytotoxicity was evaluated by a CytoTox 96^®^ lactate dehydrogenase (LDH) test (Promega Corporation, Madison, WI, USA). The cell morphology was investigated by epi-fluorescence microscopy under a Leica DM6 B epifluorescence microscope (Leica Camera AG, Wetzlar, Germany), equipped with a Leica DFC 9000 GT camera and appropriate fluorescence objectives and filters. The actin cytoskeleton was stained with Alexa Fluor™ 546 phalloidin (Invitrogen Corporation, Waltham, MA, USA), whilst the cell nuclei were counterstained with 4′,6-diamidino-2-phenylindole (DAPI) (Sigma-Aldrich, St. Louis, MO, USA). The cell culture procedures and the assay protocols can be found in full detail in previous studies by the group [45,46,47,48]. The in vitro screen testing of the sintered piezoceramics’ cytocompatibility was performed after 36 h of cell culturing.

#### 2.4.2. Macro-Porous Scaffolds

In the case of scaffolds, the cytocompatibility assessment was performed following a comprehensive, recently suggested procedure [25], which can provide a more accurate and all-encompassing picture of their biological performance, with little room for error. Namely, (i) the cell proliferation was assessed by three independent approaches (i.e., MTS, LDH, and acridine orange (AO) tests—thus inferring both the intracellular enzymes and nucleic acids paths); (ii) cell death (by an LDH cytotoxicity test); and (iii) cell morphology by fluorescence microscopy (revealing the actin cytoskeleton and nuclei of the cells, stained with Alexa Fluor™ 546 phalloidin and DAPI, respectively) and FE-SEM analyses. The in vitro biological assays of the scaffolds were performed after 14 days of cell culturing. The complete in vitro biological protocol is presented extensively in Ref. [25].

#### 2.4.3. Statistical Significance Analysis

All experiments were performed at least in triplicate. The statistical analyses in the case of the in vitro biological tests were performed, depending on the case (i.e., for two or more populations) by a two-tailed distribution unequal variance Student’s *t*-test or by a one-way ANOVA multiple analysis comparison followed by a Tukey’s post hoc test. The GraphPad Prism v.9.4.1 software (GraphPad Software, San Diego, CA, USA) was used for this purpose. A *p*-value ≤ 0.05 was considered statistically significant.

## 3. Results and Discussion

### 3.1. Multi-Parametric Analysis of the Sintered Disks

#### 3.1.1. Structural, Density and Morphological Evaluation

**Figure 1:**
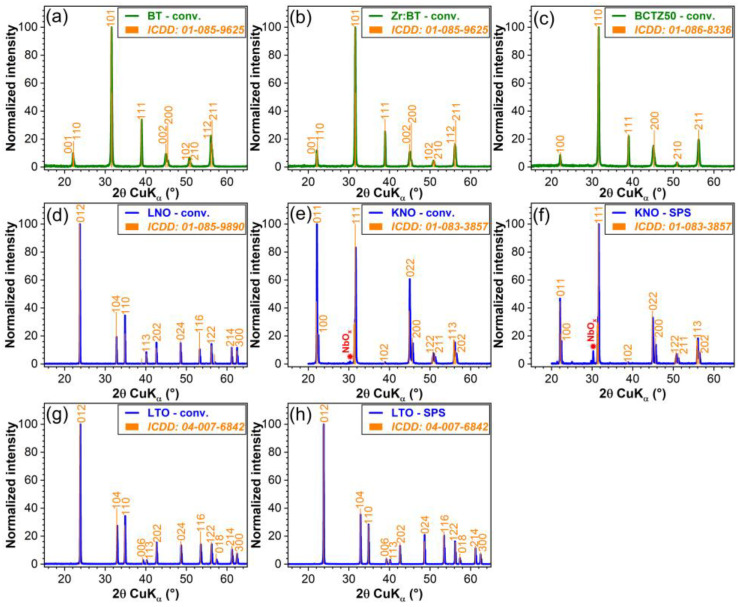
XRD diagrams of sintered ceramics: (**a**) BT; (**b**) Zr:BT; (**c**) BCTZ50; (**d**) LNO; (**e**,**f**) KNO; and (**g**,**h**) LTO, sintered conventionally (conv.) (**a–e**,**g**) or by (**f**,**h**) SPS. The ICDD reference diffraction lines of each targeted phase are presented (in orange colour) alongside the corresponding sample XRD pattern.

**Figure 2:**
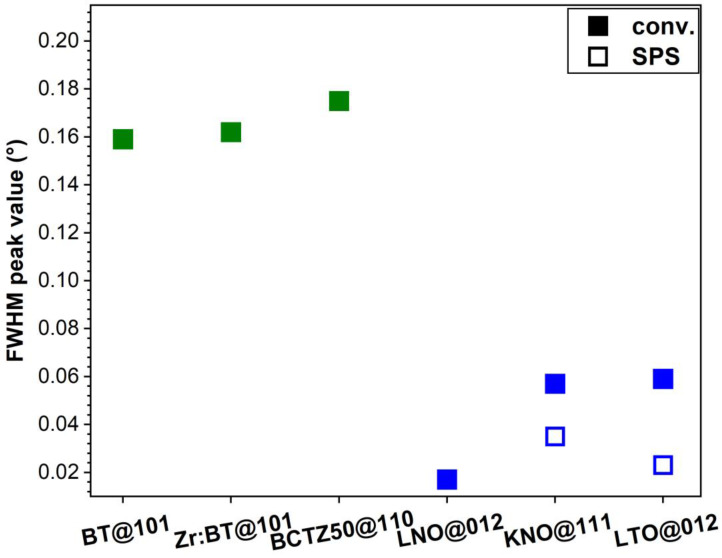
FWHM values of the most intense diffraction peak of each ceramic sample, sintered either conventionally (conv.) (solid symbols) or by SPS (hollow symbols).

The structure of the sintered ceramics was investigated by XRD. The diffractograms of each type of sample are displayed comparatively in Figure 1, with respect to the reference files (ICDD-PDF4+ database, 2022 edition) of the targeted crystalline phases (presented as superimposed sticks). With the sole exception of the KNO specimens, all the other sintered ceramics elicited a mono-phasic structure: BT—tetragonal (ICDD: 01-085-9625); Zr:BT—tetragonal (ICDD: 01-085-9625); BCTZ50—tetragonal (ICDD: 01-086-8336); LNO—rhombohedral (ICDD: 01-085-9890); and LTO—rhombohedral (ICDD: 04-007-6842). In the case of KNO, irrespective of the sintering method, besides the major KNbO_3_ orthorhombic constituent (ICDD: 01-083-3857), niobium suboxides (e.g., NbO_0.76_, ICDD: 04-011-9825) were found as ubiquitous minor residual phases. The excellent crystallinity of all sintered piezoceramics—markedly, for the BT, Zr:BT and BCTZ50 ones, showing similar crystalline quality—was demonstrated by the sharp diffraction peaks with reduced FWHM values (Figure 2). Noteworthy, the spark plasma sintering of KNO and LTO ceramics led to an improved crystalline quality (Figure 2).

**Figure 3:**
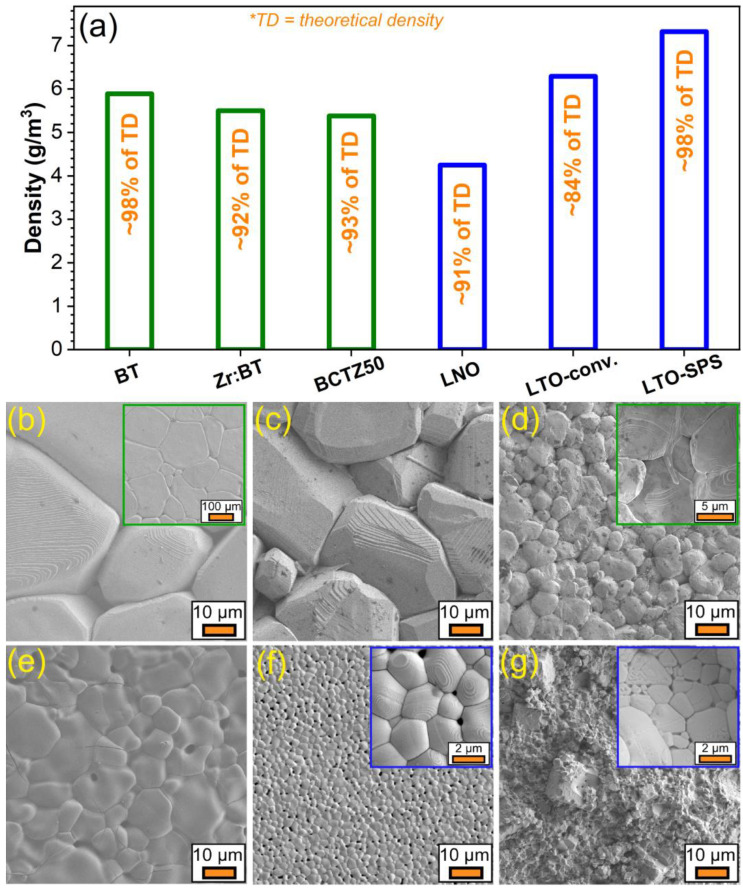
(**a**) Density values of the sintered piezoceramic materials. FE-SEM morphology of the conventionally sintered (**b**) BT; (**c**) Zr:BT; (**d**) BCTZ50; (**e**) LNO; and (**f**) LTO and (**g**) spark plasma sintered LTO ceramic disks. Insets: FE-SEM images collected at: lower magnification in the case of (**b**) BT, to enable the visualization of the general morphology of the sintered disks, and at higher magnification for the finer grained materials—(**d**) BCTZ50; and both (**f**) conventionally and (**g**) spark plasma sintered LTO, to disclose the morphology and size of the constituting grains.

The ceramic pellets, sintered by different procedures (see Table 1), presented in most of the cases an excellent densification, with values generally exceeding 90% (Figure 3a). The density values of the BT, Zr:BT, BCTZ50, LNO and LTO ceramics conventionally sintered were, respectively, ~98, 92, 93, 91 and 84% of the theoretical density (TD) of these materials (Figure 3a). In the case of LTO, a better densification (i.e., ~98%) was obtained by SPS, in good agreement with the XRD results (Figure 2). However, despite this remarkable improvement, realistically (practically) speaking, the SPS process would be difficult to implement in the production chain of macro-porous scaffolds. This is why the in vitro biological assays were performed only for the conventionally sintered LTO samples.

Characteristic FE-SEM images of the sintered piezoceramics are presented in Figure 3b–e. Their morphology was found to be in good agreement with the density evaluation, revealing compact micro-structures, constituted of intimately packed polyhedral grains. The BT and Zr:BT samples had the largest grains, with dimensions stretching from ~25 µm to more than 150 µm in the case of undoped BT, and from ~15 to ~50 µm for the Zr:BT (Figure 3b,c). The BCTZ50 and LNO samples were composed of grains with sizes in the range of ~3–20 µm (Figure 3d,e). The lowest grain sizes were noticed for the LTO ceramics, which, in the case of conventional sintering, were situated in the narrow range of ~1–3 µm, whilst in the case of the SPS ones, most grains were confined as well to the ~1–3 µm dimensional domain, but with infrequent cases of smaller (<1 µm) and larger (~12 µm) grains.

#### 3.1.2. Dielectric and Piezoelectric Properties

**Figure 4:**
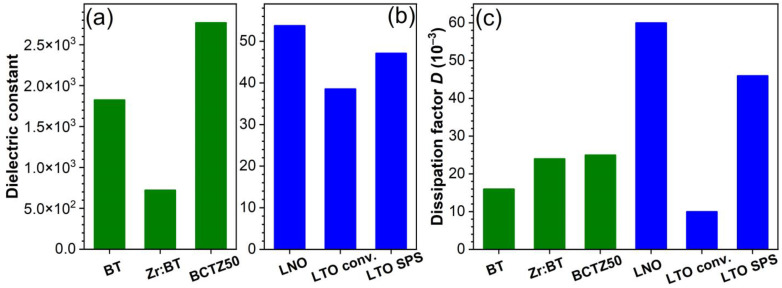
(**a**,**b**) Dielectric constant of the: (**a**) BT, Zr:BT, BCTZ50 and (**b**) LNO and LTO sintered ceramics. (**c**) Dielectric losses (dissipation factors) of the BT, Zr:BT, BCTZ50, LNO and LTO ceramics.

The dielectric properties of the sintered ceramic pellets were evaluated by capacitance and dielectric loss measurements; additionally, the dielectric features were assessed by the subsequent determination of the dielectric constant (ε). The recorded dielectric constant and dielectric loss (dissipation factors) values are presented in Figure 4a–c, respectively. The dielectric constant of BCTZ50 was found to be significantly higher, i.e., by 1.5 and 3 times compared to that of BT and Zr:BT, respectively. This behaviour can be induced by the proximity of the narrow transition between three ferroelectric phases with rhombohedral, orthorhombic and tetragonal crystal symmetries, with a very low energy barrier for the polarization rotation and lattice distortion, from one phase to another, thereby, resulting in the considerable enhancement of the dielectric and piezoelectric properties [49,50,51]. Comparable dielectric losses were delivered by the BT, Zr:BT and BCTZ50 sintered ceramics. The dielectric constants of LNO and LTO (Figure 4b) were substantially lower than those of BT-based materials (i.e., by ~40 times), while their dissipation factors were two or even three times higher than the latter ones (Figure 4c), with the exception of the conventionally sintered LTO specimens. This could be the result of the low grain size (~1–3 µm) (Figure 3f) and an increased concentration of vacancies, typical of SPS processes (employing very short sintering times and lacking an oxygenated atmosphere), which can decrease the dielectric constant (by domain refinement and weakening of the long-range ferroelectric interactions [52,53]) and enhance the electrical conductivity.

**Figure 5:**
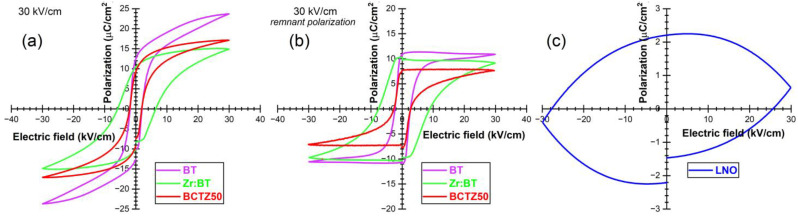
The (**a**) total and (**b**) remnant polarization vs. electric field of BT, Zr:BT and BCTZ50. (**c**) Total polarization vs. electric field in the case of LNO.

The hysteresis loops acquired for the BT, Zr:BT and BCZT50 samples are shown in Figure 5a. Similar polarization–electrical (P–E) field characteristics were recorded for the BT and BCTZ50 samples, eliciting sharp loops with an S-shape and small coercive fields (below 3 kV/cm), unlike the loops of the Zr:BT ceramics, which were significantly wider and had higher coercive fields (6 kV/cm). BT yielded the highest polarization (P_max_ = 25 µC/cm^2^). The remnant component of the polarization (P_R_), which is due to electric dipoles that remain aligned along the electric field direction, retaining their orientation even after the field was removed, is presented in Figure 5b. The rectangular shapes of the hysteresis loops indicated the excellent ferroelectric features of the investigated BT-based materials. The highest P_R_ value was recorded for BT (11.4 µC/cm^2^), followed by Zr:BT (9.8 µC/cm^2^) and BCTZ50 (7.7 µC/cm^2^), all noteworthy quality responses for this class of materials. The assessment of the remnant polarization was found very useful for further determinations since it can be proportionally correlated with the poling degree of the ferroelectric ceramics, found at the core of the piezoelectric investigations. Poling is the process of aligning the electric dipoles mainly along the electric field direction, which generates a macroscopic polarization of the ceramic or polycrystal and a piezoelectric response of the material, that, in other conditions, will not exist. The P–E loop of the LNO sintered ceramic (Figure 5c) is completely different from those recorded in the case of BT-based ceramics, having a rather typical aspect to materials with a high electrical conductivity. Similar P–E loops were registered for the LTO ceramics as well, regardless of the sintering procedure (data not shown). Such P–E loops were also reported elsewhere [54], with its authors considering it as a performant ferroelectric behaviour, while disregarding the high conductivity of the material. A more realistic approach was presented in reference [55], where a similar P–E loop, with extremely low P_R_, less than 0.1 µC/cm^2^ was attributed to a relatively high leakage caused by the lithium–oxygen diffusion near the surface, during the sintering of the La (5 mol%) doped LTO ceramic. This behaviour could be the effect of the non-stoichiometric compositions or structural defects occurring during the sintering process and caused by the evaporation of Li.

**Figure 6:**
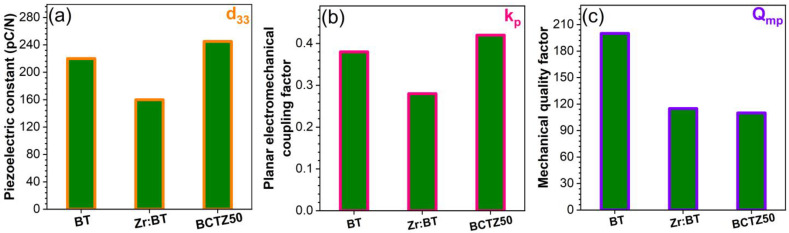
(**a**) Longitudinal piezoelectric constant d_33_; (**b**) planar coupling factor k_p_; and (**c**) mechanical quality factor Q_mp_ of the BT, Zr:BT-Zr and BCTZ50 sintered ceramics.

The piezoelectric properties were studied in both the quasi-static and the resonant regimes. The quasi-static regime consists in applying a variable compression force in the direction of poling (perpendicular to the surface of the ceramic discs) by a piezometer. Such equipment enables the measurement of the electric charge generated by the direct piezoelectric effect and the evaluation of the longitudinal piezoelectric constant (d_33_). The best d_33_ values were obtained for the pure BT and BCTZ50 sintered ceramics (Figure 6a). The resonant regime consisted in applying a low-amplitude variable-frequency alternating electric field to the piezoelectric resonator (i.e., sintered piezoceramic disk), which starts to vibrate, due to the inverse piezoelectric effect. The complex impedance spectrum of this resonator, in the frequency range corresponding to the radial resonance–antiresonance vibration mode, allows for the determination of the piezoelectric and electromechanical constants specific to that mode. The piezoelectric response of the resonator was assessed by determining, by specific calculations, the planar electromechanical coupling factor (k_p_) and the mechanical quality factor (Q_mp_), whose evolution is presented comparatively in Figure 6b,c, respectively. The BT and BCTZ50 samples presented similar piezoelectric and electromechanical responses in terms of d_33_ and k_p_, with higher values corresponding to BCTZ50, mostly because of the increased degrees of freedom available to the system (including polarization), corresponding to a strongly degenerated free energy, weak polarization and lattice anisotropy, near the narrow transition between the three phases [56]. This leads to enhanced piezoelectric and dielectric coefficients, as previously discussed. The quality factor Q_mp_ had similar values for all BT-based materials. Although the LNO and LTO ceramics were also well-densified, they have not shown a measurable piezoelectric response, even when subjected to high poling electric fields (≥30 kV/cm), due to the aforementioned increased leakage.

**Figure 7:**
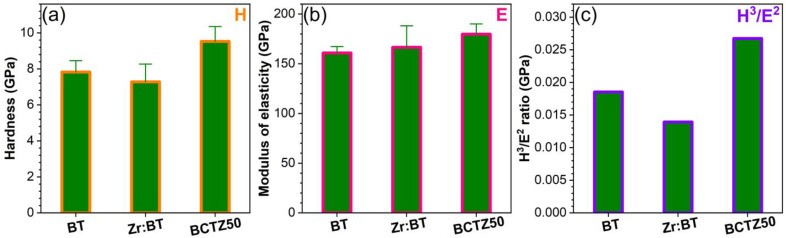
(**a**) Hardness; (**b**) modulus of elasticity; and (**c**) H^3^/E^2^ ratio (“plastic index”) determined for the BT, Zr:BT and BCTZ50 sintered ceramics based on indentation tests.

Further, only the sintered ceramics with piezoelectric response were subjected to mechanical testing for the determination of the hardness (H) and elastic modulus (E). Comparable H and E values were obtained (Figure 7a,b) for the BT-based ceramics, similar to previous literature reports [57,58]. Nevertheless, the highest values of H (~9.5 GPa) and E (~180 GPa) obtained in the case of BCTZ50 can be emphasised. These mechanical traits of BCTZ50 led to the highest H^3^/E^2^ ratio (Figure 7c), which gives a relative measure of a material’s ability to dissipate plastic deformation energy during mechanical loading [59], of certain importance for SBGS applications. Considering this aspect, the synthesised BCTZ50 piezoceramic holds superior mechanical promise with respect to the other two BT-based materials.

#### 3.1.3. In Vitro (pH and Cell Compatibility) Response

**Figure 8:**
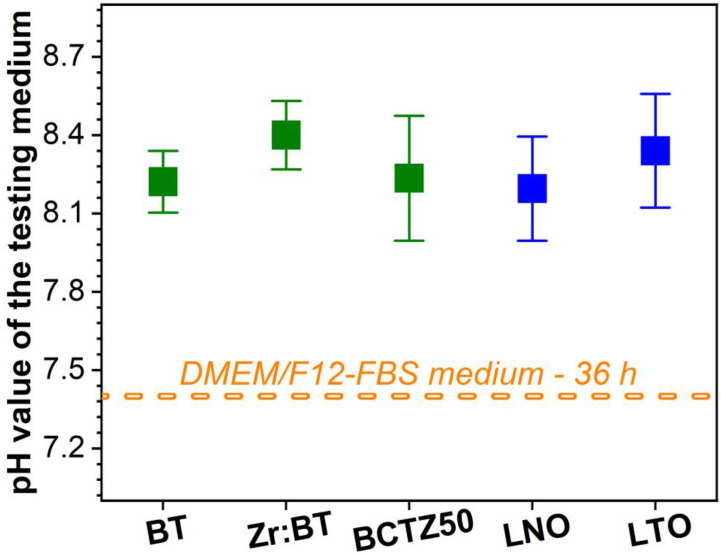
pH value of the DMEM/F12-FBS cell culture medium after 36 h of incubation in the presence of BT, Zr:BT, BCTZ50, LNO and LTO sintered ceramics.

The average pH values of the DMEM/F12-FBS cell culture medium incubated for 36 h in the presence of the sintered ceramic specimens are shown in Figure 8. All the tested ceramics had an alkalinization tendency, slightly shifting the pH of the cell culture medium towards values in the range of ~8.2–8.4. No statistically significant differences were recorded between the tested ceramics (*p* > 0.05, one-way ANOVA followed by Tukey’s post hoc test).

**Figure 9:**
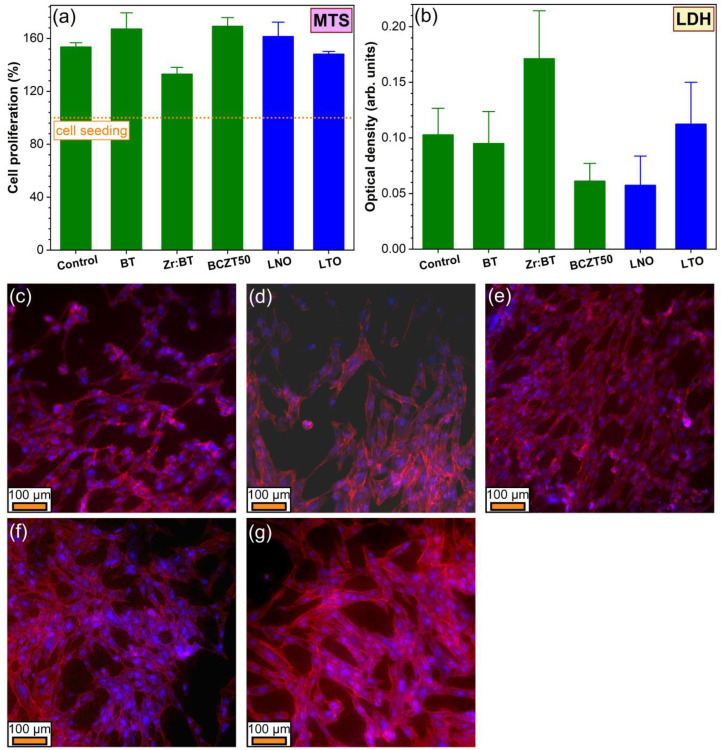
(**a**) hFOB 1.19 cell proliferation of the sintered ceramics, as assessed by an MTS assay performed after 36 h of culturing; (**b**) cytotoxicity of the investigated piezoceramics, as inferred by an LDH test after 36 h of culturing; (**c**–**g**) morphology of hFOB 1.19 cells after 36 h of culturing on the surface of the (**c**) BT; (**d**) Zr:BT; (**e**) BCTZ50; (**f**) LNO; and (**g**) LTO disks, as evidenced by epifluorescence microscopy. The actin cytoskeleton is stained with red (Alexa Fluor™ 546 phalloidin), whilst the cell nuclei are counterstained with blue (DAPI).

The cytocompatibility of the as-sintered ceramics has been assessed by a suite of in vitro tests that revealed the proliferation, death and morphological traits of the surface-grown osteoblast cells. Remarkably, all ceramics induced an excellent proliferation of hFOB 1.19 cells, having mean values comparable to or even higher than those of the biological control (i.e., standard tissue culture grade polystyrene surface), which was more pronounced in the case of the pure BT and BCTZ50 ceramics (Figure 9a). However, statistically significant differences (*p* < 0.05, one-way ANOVA followed by Tukey’s post hoc test) were observed only for Zr:BT which elicited lower cell proliferation performances when compared to the biological control and the BT, BCTZ50 and LNO samples. 

In terms of cell death, the lowest values of LDH activity were yielded by the BCTZ50 and LNO samples (Figure 9b), a testimony of their very reduced cytotoxicity. The highest cytotoxic index was generated by the Zr:BT sintered ceramics (statistically significant differences with respect to the control and the other scrutinised ceramic materials, *p* < 0.05, one-way ANOVA followed by Tukey’s post hoc test), in good agreement with the cell proliferation observations (Figure 9a). This lesser in vitro biological performance of Zr:BT, although not worrying, since the hFOB cells proliferated compared to their seeding number, could be ascribed to the higher released concentration of Ba in the cell culture medium (i.e., ~70 mg/L) with respect to the other BT-based ceramics (which leached a Ba concentration of ~17–20 mg/L) (as determined by ICP-MS, data not shown, statistics on small sample batch sizes). It should be emphasised that the concentration levels of Ba and Li ions (situated in the range of ~15–18 mg/L) released in the culture medium by the tested ceramics are far below the reported toxicity limits [60,61], which should make these sintered ceramics biologically safe and therefore promising options for further development of bone regeneration applications (including the SBGS constructs). It is important to specify that Zr, Ti, Nb and Ta are well-known bioinert elements, either constituting self-standing bioceramics (i.e., zirconia) or being frequently incorporated in medical grade alloys or metallic implants (e.g., Ti superalloys, such as Ti6Al7Nb; Ta porous scaffolds), and do not pose toxicity concerns.

Irrespective of the type of sintered ceramic, the osteoblast cells preserved their characteristic morphology (Figure 9c–d), with the actin filaments (i.e., dynamic fibrillar constructs belonging to the cytoskeleton structure), being grouped in bundles, and spreading the cells on the tested sample surfaces. The cell nuclei presented normal shapes and sizes, without pathological chromatin condensations. Altogether, a high cytocompatibility of all tested piezoceramic materials is suggested, with the best cellular response being exhibited by the BCTZ50 samples.

By collective assessment, the best functional (electrical, mechanical and in vitro biological) response was yielded by the BCTZ50-type sintered ceramic, which confers, in addition to excellent cytocompatibility, also promising mechanical response and good piezoelectric properties, and thereby, was selected for performing further pilot tests of 3D printing by robocasting of macro-porous scaffolds, followed by preliminary compressive strength and in vitro cytocompatibility evaluations.

### 3.2. Translation to Real Biomedical Applications—Pilot Studies of BCTZ Macro-Porous Bone Scaffolds

The generation of small electric charges induced by the piezoelectric effect under biomechanical stress is predicted to signal to osteoblast cells to orientate the efforts of remodelling and healing in the direction of the applied force, leading to new bone strengthening and rendering resistance [62,63,64,65]. This created the premises for defining a new generation of macro-porous bone graft substitutes utilizing the piezoelectrical triggers for boosting bone regeneration, a subject which only recently, although rarely, started being tackled (e.g., BT [66], barium strontium titanate/β-TCP blends [67], BCTZ—simple [68] or coated with bioactive glass [69], or (K,Na)NbO_3_ [70]). Besides the outcomes of the above-presented unitary screening research of a series of relevant piezoceramics, the (i) recognised excellent piezoelectric response of BCTZ [49], as well as the (ii) possibility to further improve its piezoelectric performance by future compositional and structural engineering [71,72,73,74,75,76,77], represent supplementary legitimate reasons for advancing BCTZ for the fabrication of macro-porous scaffolds.

#### 3.2.1. Morphological Analyses

**Figure 10:**
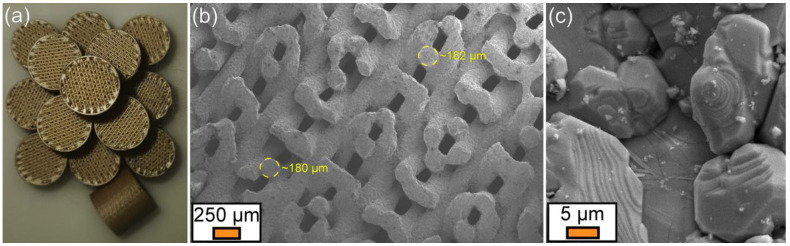
(**a**) Batch of sintered BCTZ50 scaffolds printed by robocasting. (**b**) General view of the filament arrangement and size, as evidenced by a low magnification FE-SEM image of a BCTZ50 scaffold fractured along its height. (**c**) Micro-structure of the sintered BCTZ50 filaments, as revealed by high-magnification FE-SEM analysis.

Subsequent to robocasting printing, drying, calcination and sintering, the macro-porous BCTZ50 scaffolds were found conformal (Figure 10a), suggesting the capacity of the extruded ceramic paste filaments to retain their shape and support the layers deposited on top in a subsequent 45-degrees rotated overlapping manner. This was further confirmed by FE-SEM analysis (Figure 10b), which indicated that the filaments are rectilinear and elicit a spherical cross-section, with their diameter being reduced by ~28% after sintering (from 250 to ~180 µm). The edge-to-edge inter-filament distances were ~160 µm. Based on these data, the scaffold architecture was reconstructed in the SolidWorks 3D CAD 2019 software (Dassault Systèmes, Vélizy-Villacoublay, France). This allowed empirically evaluating the macro-porosity of the designed SBGS at ~50%. The higher FE-SEM magnification analysis showed the good densification of the filaments (Figure 10c), composed of polyhedral closely packed grains with similar sizes to those observed in the case of the bulk ceramic disk samples (Figure 3d).

#### 3.2.2. Compressive Strength Performance

The BCTZ scaffolds, with a macro-porosity of ~50% after sintering, and pore sizes of ~160 µm, had compressive strength values of ~20.2 ± 6.0 MPa. The trabecular and cortical bones present values of compressive strength situated in the ranges 0.2–16 and 100–230 MPa [78,79,80], respectively, depending on age and anatomical area. The compressive strength response of our BCTZ scaffolds is situated above the prerequisites of trabecular bone applications, which makes them promising in this respect. For translation to higher load-bearing applications, cation-doping, printing geometries and sintering schedule designs need to be further explored.

#### 3.2.3. Cytocompatibility Assessments

**Figure 11:**
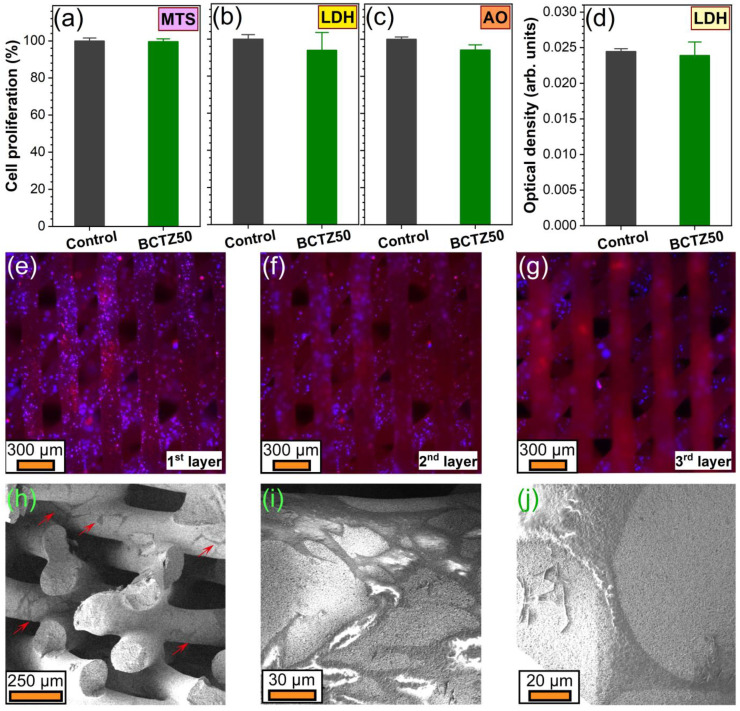
(**a**–**c**) hFOB 1.19 cell proliferation values recorded for the BCTZ50 scaffolds, determined by (**a**) MTS, (**b**) LDH and (**c**) Acridine orange (AO) tests after 14 days of culturing. (**d**) Cytotoxicity of the BCTZ50 scaffolds evaluated by an LDH test after 14 days of cell culturing. (**e**–**j**) Effective colonization (after 14 days) of the BCTZ50 scaffolds with hFOB 1.19 cells evidenced by (**e**–**g**) epi-fluorescence microscopy images focused on the first three printed layers and by (**h**) FE-SEM analysis. (**e**–**g**) The actin cytoskeleton is stained with red (Alexa Fluor™ 546 phalloidin, Thermo Fisher Scientific, Waltham, MA, USA), whilst the cell nuclei are counterstained with blue (DAPI). (**h**) Several cell bundles are indicated on the FE-SEM image with red arrows. (**i**,**j**) Morphology of hFOB 1.19 cells grown for 14 days on the surface of the filaments constituting the BCTZ50 macro-porous scaffolds, revealed by FE-SEM investigations.

If striving for a complete and trustworthy portrayal of the in vitro cytocompatibility response of complex compositional materials, such as BCTZ ceramics, a multi-test biological approach needs to be adopted. Thus, since some ions could inhibit enzymes or some materials can adsorb reaction products, it was decided to explore cell proliferation by three independent assays (tackling both the enzymatic (MTS and LDH) and nucleic acids (AO) routes). Remarkably, the BCTZ50 scaffolds delivered cell proliferation values similar to those induced by the biological control (standard cell culture dedicated surfaces) with no statistically significant differences (*p* > 0.05, two-tailed distribution unequal variance Student’s *t*-test), irrespective of the type of cell proliferation testing approach (MTS—Figure 11a, LDH—Figure 11b, and AO—Figure 11c). However, as presented in previous works [25,46], it is always advisable to simultaneously carry out cytotoxicity tests in parallel to the cell proliferation tests, as, in some cases, the cellular turnover can be increased, and thus, higher proliferation can coexist with higher cell death rates. Moreover, when working with heterogeneous cell populations that have different capacities to metabolise formazan, LDH values can account for hidden death. The BCTZ50 scaffolds yielded analogous cell death values (Figure 11d) to those registered for the biological control (*p* > 0.05, two-tailed distribution unequal variance Student’s *t*-test), thus having an insignificant intervention on the cell mortality and further highlighting the excellent cytocompatible response of these macro-porous ceramic constructs. The epi-fluorescence microscopy analyses provided definite proof that the BCTZ scaffolds were well-colonised by the osteoblast cells, these being clearly visible in large populations down to at least the third layer of the scaffolds (Figure 11e–g). This was facilitated by the long focus distance of the employed microscope objectives dedicated to biological applications. However, due to the 45-degree-rotated printed design of the scaffolds, the area to be examined decreased with each filament level down, and thus the exploration of the cell colonization of the scaffolds farther in depth than the third layer was not possible due to the depth of field limitations of this microscopy technique. Nevertheless, the FE-SEM analysis of fractured scaffolds provided further evidence that the cells were present in bundles far more in depth. The larger magnification FE-SEM images (Figure 11i,j) showed that the osteoblast cells were well-spread and attached on the surface of the BCTZ printed filaments and formed lamellipodia and filopodia, protrusive extensions critical for the cell–cell communication, as well as for the cell motility and ability to explore and penetrate into spaces. Thereby, this preliminary in vitro biological testing suite emphasised the noteworthy cytocompatibility of BCTZ50, analogous to the control, which is reassuring in view of the planned future studies aiming to assess the stem cell proliferation and differentiation/preservation of a stem cell pool of these piezoceramics in the absence and presence of relevant biomechanical stresses.

Although, without doubt promising, the path towards the implementation of BCTZ-based piezoceramics into real-world bone regeneration applications (furthermore in the case of those targeting cortical bone substitution/treatment) must be considered as only commencing, since several additional aspects and demanding requisites need to be further tackled/solved, imposing supplementary efforts from the research community. 

The piezoelectric and dielectric properties (resulting from the reduced polarization and lattice anisotropy) are known to be enhanced in the vicinity of phase transitions [50,56]. In this regard, BCTZ is a superior piezoceramic with respect to BT since it elicits improved responses at body temperature, near a “phase convergence region” between the three ferroelectric (rhombohedral, orthorhombic and tetragonal) phases and the paraelectric (cubic) phase [50], making it attractive for bone regeneration applications. Furthermore, the orthorhombic-to-tetragonal phase transition of BCTZ can be further tailored by compositional adjustments. Instead, the orthorhombic-to-tetragonal and the tetragonal-to-cubic phase transitions take place in the case of BT around 5 and 130 °C, respectively [81], making it more difficult to capitalise its full piezoelectric potential in the framework of biological applications. Besides, BCTZ yielded superior mechanical (in terms of hardness and elastic modulus) and in vitro biological performances with respect to the other tested ceramics. 

However, bone, due to its complex organic–inorganic structure, fosters hardness (0.4–0.9 GPa) and elastic modulus (10–30 GPa) properties dissimilar to those of piezoceramics [82,83]. This could lead to the advent of stress-shielding phenomena, especially when targeting cortical bone substitution/repair, having as a consequence, in the worst-case scenario, the aseptic loosening of the implanted device [84,85,86,87]. Thereby, the compatibilization of mechanical properties between SBGS constructs and host bone represents a future task of paramount importance, which might be tackled by implementing polymeric materials in composite 3D-printed scaffolds. In this respect, over the years, there have been suggestions for a series of polymeric piezoelectric materials, with noteworthy capabilities for bone regeneration. As possible candidates for designing macro-porous scaffolds based on piezoelectric composites, one can mention: collagen, silk, cellulose, chitosan, polyhydroxybutyrate (PHB), poly(L-lactide) (PLLA), polyamide-11, poly(vinylidene fluoride) (PVDF) and its co-polymers poly(vinylidene fluoride-trifluoroethylene) (PVDF-TrFE) and poly(vinylidene fluoride-hexafluoropropylene) (PVDF-HFP) [30,64,88,89,90,91]. Amongst them, PVDF-based materials are perhaps the most promising, possessing the highest piezoelectric coefficients (i.e., situated in the range of 24–38 pC/N, depending on composition), and furthermore being already tested successfully in bone-related applications in both single form [30,88,92] and coupled with piezoelectric (e.g., BT [93,94,95,96,97]) or bioactive (e.g., hydroxyapatite [98,99,100]) ceramics, both in vitro [93,94,95,98,99,100,101] and in vivo [96,97,102,103].

## 4. Conclusions

A series of lead-free piezoelectric ceramics were for the first time compared in a multi-parametric unitary study, employing analogous physico-chemical, mechanical and in vitro biological investigation techniques, aiming to unambiguously delineate a biocompatible piezoceramic material candidate for a next generation of synthetic bone graft substitutes. In this respect, the BCTZ50 ceramic was found to be endowed with a set of promising features in terms of densification by conventional sintering, dielectric constant (1.5 times higher than the next best material, i.e., undoped BT), piezoelectric properties (longitudinal piezoelectric constant (d_33_) and planar electromechanical coupling factor (k_p_)), mechanical response (hardness of ~8.4 GPa and elastic modulus of ~153 MPa, leading to the highest H^3^/E^2^ ratio, which suggested the material’s ability to dissipate plastic deformation energy during mechanical loading) and in vitro performance (in terms of both cell proliferation and cytotoxicity).

The BCTZ50 ceramic was further successfully implemented into macro-porous scaffolds in the framework of a series of pilot robocasting printing experiments, which were followed by preliminary mechanical and in vitro biological studies. Conformal BCTZ50 scaffolds were fabricated and sintered at 1500 °C, having a macro-porosity of ~50%. The highly porous scaffolds possessed a compressive strength of ~20 MPa, superior to trabecular bone. The BCTZ constructs were uniformly colonised by osteoblast cells. Remarkably, their cytocompatible response (tested by a suite of cell proliferation, cell death and cell morphology analyses) was analogous to that of the biological control. Altogether, promising prerequisites were settled for the future development and complex testing of a possible new generation of synthetic bone graft substitutes, which could make use of the piezoelectric effect as an osteogenic boosting trigger. 

Future studies will be envisioned aiming to (*i*) improve the mechanical properties by adopting different scaffold geometries and pore size and distribution, as well as BCTZ cation doping and/or coupling with piezoelectric polymers, and (*ii*) assess the stem cell differentiation/preservation of a stem cell pool in the absence and presence of appropriate biomechanical stresses.

## Figures and Tables

**Table 1:** Optimised sintering conditions for fabricating dense piezoelectric ceramics.

CERAMIC DISKS DENSIFIED THROUGH CONVENTIONAL SINTERING PROCESS					
Material	Calcination Temperature (°C)	Pressing Force (kg/cm^2^)	Atmosphere	Sintering Temperature (°C)	Sintering Duration (h)
BT	1000	200	Air	1300	3
Zr:BT	1000	150	Air	1300	3
BCTZ50	1350	80	Air	1550	4
LNO	800	80	Air	1175 *	2
KNO	700	50	Air	1000 *	2
LTO	700	40	Air	1375	3
**CERAMIC DISKS DENSIFIED THROUGH SPARK PLASMA SINTERING (SPS)**					
**Material**	**Calcination** **temperature** **(°C)**	**Pressing force (MPa)**	**Atmosphere**	**Sintering** **temperature** **(°C)**	**Sintering** **duration (min)**
KNO	600	60	Vacuum, 40 hPa	900	3
LTO	800	60	Vacuum, 40 hPa	1150	3

* At higher sintering temperatures these specific ceramics started melting.

## Data Availability

Raw data can be made available per request to the corresponding author.

## References

[1] Mishra R., Bishop T., Valerio I.L., Fisher J.P., Dean D. (2016). The potential impact of bone tissue engineering in the clinic. Regen. Med..

[2] Polo-Corrales L., Latorre-Esteves M., Ramirez-Vick J.E. (2014). Scaffold design for bone regeneration. J. Nanosci. Nanotechnol..

[3] De Witte T.-M., Fratila-Apachitei L.E., Zadpoor A.A., Peppas N.A. (2018). Bone tissue engineering via growth factor delivery: From scaffolds to complex matrices. Regen. Biomater..

[4] Yang Y., Wang G., Liang H., Gao C., Peng S., Shen L., Shuai C. (2018). Additive manufacturing of bone scaffolds. Int. J. Bioprinting.

[5] World Health Organization Life Expencancy and Healthy Life Expectancy. https://www.who.int/data/gho/data/themes/mortality-and-global-health-estimates/ghe-life-expectancy-and-healthy-life-expectancy.

[6] Ebrahimi M., Botelho M.G., Dorozhkin S.V. (2017). Biphasic calcium phosphates bioceramics (HA/TCP): Concept, physicochemical properties and the impact of standardization of study protocols in biomaterials research. Mater. Sci. Eng. C.

[7] Montero J., Becerro A., Pardal-Peláez B., Quispe-López N., Blanco J.-F., Gómez-Polo C. (2021). Main 3D manufacturing techniques for customized bone substitutes. A systematic review. Materials.

[8] Pedrero S.G., Llamas-Sillero P., Serrano-López J. (2021). A multidisciplinary journey towards bone tissue engineering. Materials.

[9] Mohd N., Razali M., Ghazali M.J., Abu Kasim N.H. (2022). 3D-printed hydroxyapatite and tricalcium phosphates-based scaffolds for alveolar bone regeneration in animal models: A scoping review. Materials.

[10] Zaszczyńska A., Moczulska-Heljak M., Gradys A., Sajkiewicz P. (2021). Advances in 3D printing for tissue engineering. Materials.

[11] Hannink G., Arts J.J.C. (2011). Bioresorbability, porosity and mechanical strength of bone substitutes: What is optimal for bone regeneration?. Injury.

[12] Grand View Research Bone Grafts and Substitutes Market Size, Share & Trends Analysis. https://www.grandviewresearch.com/industry-analysis/bone-grafts-substitutes-market.

[13] Precedence Research Bone Grafts and Substitutes Market. https://www.precedenceresearch.com/bone-grafts-and-substitutes-market.

[14] Ghassemi T., Shahroodi A., Ebrahimzadeh M.H., Mousavian A., Movaffagh J., Moradi A. (2018). Current concepts in scaffolding for bone tissue engineering. Arch. Bone Jt. Surg..

[15] Wang W., Yeung K.W.K. (2017). Bone grafts and biomaterials substitutes for bone defect repair: A review. Bioact. Mater..

[16] Schmidt A.H. (2021). Autologous bone graft: Is it still the gold standard?. Injury.

[17] Amini Z., Lari R. (2021). A systematic review of decellularized allograft and xenograft-derived scaffolds in bone tissue regeneration. Tissue Cell.

[18] Bracey D.N., Cignetti N.E., Jinnah A.H., Stone A.V., Gyr B.M., Whitlock P.W., Scott A.T. (2020). Bone xenotransplantation: A review of the history, orthopedic clinical literature, and a single-center case series. Xenotransplantation.

[19] Zhao R., Yang R., Cooper P.R., Khurshid Z., Shavandi A., Ratnayake J. (2021). Bone grafts and substitutes in dentistry: A review of current trends and developments. Molecules.

[20] Roseti L., Parisi V., Petretta M., Cavallo C., Desando G., Bartolotti I., Grigolo B. (2017). Scaffolds for bone tissue engineering: State of the art and new perspectives. Mater. Sci. Eng. C.

[21] Yan Y., Chen H., Zhang H., Guo C., Yang K., Chen K., Cheng R., Qian N., Sandler N., Zhang Y.S. (2019). Vascularized 3D printed scaffolds for promoting bone regeneration. Biomaterials.

[22] Gritsch L., Maqbool M., Mouriño V., Ciraldo F.E., Cresswell M., Jackson P.R., Lovell C., Boccaccini A.R. (2019). Chitosan/hydroxyapatite composite bone tissue engineering scaffolds with dual and decoupled therapeutic ion delivery: Copper and strontium. J. Mater. Chem. B.

[23] Rizwan M., Hamdi M., Basirun W.J. (2017). Bioglass^®^ 45S5-based composites for bone tissue engineering and functional applications. J. Biomed. Mater. Res. Part A.

[24] Lu J., Yu H., Chen C. (2018). Biological properties of calcium phosphate biomaterials for bone repair: A review. RSC Adv..

[25] Besleaga C., Nan B., Popa A.-C., Balescu L.M., Nedelcu L., Neto A.S., Pasuk I., Leonat L., Popescu-Pelin G., Ferreira J.M.F. (2022). Sr and Mg doped bi-phasic calcium phosphate macroporous bone graft substitutes fabricated by robocasting: A structural and cytocompatibility assessment. J. Funct. Biomater..

[26] Mirzaali M.J., Schwiedrzik J.J., Thaiwichai S., Best J.P., Michler J., Zysset P.K., Wolfram U. (2016). Mechanical properties of cortical bone and their relationships with age, gender, composition and microindentation properties in the elderly. Bone.

[27] Li J., Bao Q., Chen S., Liu H., Feng J., Qin H., Li A., Liu D., Shen Y., Zhao Y. (2017). Different bone remodeling levels of trabecular and cortical bone in response to changes in Wnt/β-catenin signaling in mice. J. Orthop. Res..

[28] Saito Y., Takao H., Tani T., Nonoyama T., Takatori K., Homma T., Nagaya T., Nakamura M. (2004). Lead-free piezoceramics. Nature.

[29] Ioachim A., Alexandru H.V., Berbecaru C., Antohe S., Stanculescu F., Banciu M.G., Toacsen M.I., Nedelcu L., Ghetu D., Dutu A. (2006). Dopant influence on BST ferroelectric solid solutions family. Mater. Sci. Eng. C.

[30] Jacob J., More N., Kalia K., Kapusetti G. (2018). Piezoelectric smart biomaterials for bone and cartilage tissue engineering. Inflamm. Regen..

[31] Jarkov V., Allan S.J., Bowen C., Khanbareh H. (2022). Piezoelectric materials and systems for tissue engineering and implantable energy harvesting devices for biomedical applications. Int. Mater. Rev..

[32] Fukada E., Yasuda I. (1957). On the piezoelectric effect of bone. J. Phys. Soc. Jpn..

[33] Ulstrup A.K. (2008). Biomechanical concepts of fracture healing in weight-bearing long bones. Acta Orthop. Belg..

[34] Uto Y., Kuroshima S., Nakano T., Ishimoto T., Inaba N., Uchida Y., Sawase T. (2017). Effects of mechanical repetitive load on bone quality around implants in rat maxillae. PLoS ONE.

[35] Yu P., Ning C., Zhang Y., Tan G., Lin Z., Liu S., Wang X., Yang H., Li K., Yi X. (2017). Bone-inspired spatially specific piezoelectricity induces bone regeneration. Theranostics.

[36] Vilarinho P.M., Barroca N., Zlotnik S., Félix P., Fernandes M.H. (2014). Are lithium niobate (LiNbO_3_) and lithium tantalate (LiTaO_3_) ferroelectrics bioactive?. Mater. Sci. Eng. C.

[37] Carville N.C., Collins L., Manzo M., Gallo K., Lukasz B.I., McKayed K.K., Simpson J.C., Rodriguez B.J. (2015). Biocompatibility of ferroelectric lithium niobate and the influence of polarization charge on osteoblast proliferation and function. J. Biomed. Mater. Res. Part A.

[38] Ciofani G., Danti S., D’Alessandro D., Moscato S., Petrini M., Menciassi A. (2010). Barium titanate nanoparticles: Highly cytocompatible dispersions in glycol-chitosan and doxorubicin complexes for cancer therapy. Nanoscale Res. Lett..

[39] Candito M., Simoni E., Gentilin E., Martini A., Marioni G., Danti S., Astolfi L. (2022). Neuron compatibility and antioxidant activity of barium titanate and lithium niobate nanoparticles. Int. J. Mol. Sci..

[40] Saranya K., Thirupathi Kumara Raja S., Subhasree R.S., Gnanamani A., Das S.K., Rajendran N. (2017). Fabrication of nanoporous sodium niobate coating on 316L SS for orthopaedics. Ceram. Int..

[41] Yu S.W., Kuo S.T., Tuan W.H., Tsai Y.Y., Wang S.F. (2012). Cytotoxicity and degradation behavior of potassium sodium niobate piezoelectric ceramics. Ceram. Int..

[42] Mancuso E., Shah L., Jindal S., Serenelli C., Tsikriteas Z.M., Khanbareh H., Tirella A. (2021). Additively manufactured BaTiO_3_ composite scaffolds: A novel strategy for load bearing bone tissue engineering applications. Mater. Sci. Eng. C.

[43] Shimada S., Kodaira K., Matsushita T. (1984). Sintering LiTaO_3_ and KTaO_3_ with the aid of manganese oxide. J. Mater. Sci..

[44] Oliver W.C., Pharr G.M. (1992). An improved technique for determining hardness and elastic modulus using load and displacement sensing indentation experiments. J. Mater. Res..

[45] Popa A.C., Marques V.M.F., Stan G.E., Husanu M.A., Galca A.C., Ghica C., Tulyaganov D.U., Lemos A.F., Ferreira J.M.F. (2014). Nanomechanical characterization of bioglass films synthesized by magnetron sputtering. Thin Solid Film..

[46] Popa A.C., Stan G.E., Besleaga C., Ion L., Maraloiu V.A., Tulyaganov D.U., Ferreira J.M.F. (2016). Submicrometer hollow bioglass cones deposited by radio frequency magnetron sputtering: Formation mechanism, properties, and prospective biomedical applications. ACS Appl. Mater. Interfaces.

[47] Besleaga C., Dumitru V., Trinca L.M., Popa A.C., Negrila C.C., Kołodziejczyk Ł., Luculescu C.R., Ionescu G.C., Ripeanu R.G., Vladescu A. (2017). Mechanical, corrosion and biological properties of room-temperature sputtered aluminum nitride films with dissimilar nanostructure. Nanomaterials.

[48] Stan G.E., Tite T., Popa A.-C., Chirica I.M., Negrila C.C., Besleaga C., Zgura I., Sergentu A.C., Popescu-Pelin G., Cristea D. (2020). The beneficial mechanical and biological outcomes of thin copper-gallium doped silica-rich bio-active glass implant-type coatings. Coatings.

[49] Liu W., Ren X. (2009). Large piezoelectric effect in Pb-free ceramics. Phys. Rev. Lett..

[50] Keeble D.S., Benabdallah F., Thomas P.A., Maglione M., Kreisel J. (2013). Revised structural phase diagram of (Ba_0.7_Ca_0.3_TiO_3_)-(BaZr_0.2_Ti_0.8_O_3_). Appl. Phys. Lett..

[51] Hao J., Bai W., Li W., Zhai J. (2012). Correlation between the microstructure and electrical properties in high-performance (Ba_0.85_Ca_0.15_)(Zr_0.1_Ti_0.9_)O_3_ lead-free piezoelectric ceramics. J. Am. Ceram. Soc..

[52] Cao W., Randall C.A. (1996). Grain size and domain size relations in bulk ceramic ferroelectric materials. J. Phys. Chem. Solids.

[53] Arlt G. (1990). The influence of microstructure on the properties of ferroelectric ceramics. Ferroelectrics.

[54] Yang T., Liu Y.G., Zhang L., Hu M.L., Yang Q., Huang Z.H., Fang M.H. (2014). Powder synthesis and properties of LiTaO_3_ ceramics. Adv. Powder Technol..

[55] Diaz-Moreno C.A., Ding Y., Portelles J., Heiras J., Macias A.H., Syeed A., Paez A., Li C., López J., Wicker R. (2018). Optical properties of ferroelectric lanthanum lithium niobate. Ceram. Int..

[56] Damjanovic D., Biancoli A., Batooli L., Vahabzadeh A., Trodahl J. (2012). Elastic, dielectric, and piezoelectric anomalies and Raman spectroscopy of 0.5Ba(Ti_0.8_Zr_0.2_)O_3_-0.5(Ba_0.7_Ca_0.3_)TiO_3_. Appl. Phys. Lett..

[57] Trzepiecinski T., Gromada M. (2018). Characterization of mechanical properties of barium titanate ceramics with different grain sizes. Mater. Sci..

[58] Coondoo I., Panwar N., Alikin D., Bdikin I., Islam S.S., Turygin A., Shur V.Y., Kholkin A.L. (2018). A comparative study of structural and electrical properties in lead-free BCZT ceramics: Influence of the synthesis method. Acta Mater..

[59] Tsui T.Y., Pharr G.M., Oliver W.C., Bhatia C.S., White R.L., Anders S., Anders A., Brown I.G. (1995). Nanoindentation and nanoscratching of hard carbon coatings for magnetic disks. MRS Proc..

[60] Tite T., Popa A.C., Balescu L.M., Bogdan I.M., Pasuk I., Ferreira J.M.F., Stan G.E. (2018). Cationic substitutions in hydroxyapatite: Current status of the derived biofunctional effects and their in vitro interrogation methods. Materials.

[61] Toxicological Profile for Barium and Barium Compounds. https://www.atsdr.cdc.gov/toxprofiles/tp24-c2.pdf.

[62] Bansod Y.D., Kebbach M., Kluess D., Bader R., van Rienen U. (2021). Finite element analysis of bone remodelling with piezoelectric effects using an open-source framework. Biomech. Model. Mechanobiol..

[63] Bansod Y.D., Kebbach M., Kluess D., Bader R., van Rienen U. (2021). Computational analysis of bone remodeling in the proximal tibia under electrical stimulation considering the piezoelectric properties. Front. Bioeng. Biotechnol..

[64] Carter A., Popowski K., Cheng K., Greenbaum A., Ligler F.S., Moatti A. (2021). Enhancement of bone regeneration through the converse piezoelectric effect. A novel approach for applying mechanical stimulation. Bioelectricity.

[65] Fernández J.R., García-Aznar J.M., Martínez R. (2012). Piezoelectricity could predict sites of formation/resorption in bone remodelling and modelling. J. Theor. Biol..

[66] Schult M., Buckow E., Seitz H. (2016). Experimental studies on 3D printing of barium titanate ceramics for medical applications. Curr. Dir. Biomed. Eng..

[67] Tariverdian T., Behnamghader A., Brouki Milan P., Barzegar-Bafrooei H., Mozafari M. (2019). 3D-printed barium strontium titanate-based piezoelectric scaffolds for bone tissue engineering. Ceram. Int..

[68] Nan B., Olhero S., Pinho R., Vilarinho P.M., Button T.W., Ferreira J.M.F. (2019). Direct ink writing of macroporous lead-free piezoelectric Ba_0.85_Ca_0.15_Zr_0.1_Ti_0.9_O_3_. J. Am. Ceram. Soc..

[69] Sugimoto H., Biggemann J., Fey T., Singh P., Khare D., Dubey A.K., Kakimoto K. (2021). Lead-free piezoelectric (Ba,Ca)(Ti,Zr)O_3_ scaffolds for enhanced antibacterial property. Mater. Lett..

[70] Li Y., Li L., Li B. (2015). Direct ink writing of three-dimensional (K, Na)NbO_3_-based piezoelectric ceramics. Materials.

[71] Yang Z., Fu J., Xu Y., Zuo R. (2021). Field-insensitive giant dynamic piezoelectric response and its structural origin in (Ba,Ca)(Ti,Zr)O_3_ tetragonal-orthorhombic phase-boundary ceramics. J. Eur. Ceram. Soc..

[72] Liu Y., Zhang H., Shi W., Wang Q., jiang G., Yang B., Cao W., Tan J. (2022). Ultrahigh strain in textured BCZT-based lead-free ceramics with CuO sintering agent. J. Mater. Sci. Technol..

[73] Hayati R., Bahrevar M.A., Ganjkhanlou Y., Rojas V., Koruza J. (2019). Electromechanical properties of Ce-doped (Ba_0.85_Ca_0.15_)(Zr_0.1_Ti_0.9_)O_3_ lead-free piezoceramics. J. Adv. Ceram..

[74] Zhang Q., Cai W., Li Q., Gao R., Chen G., Deng X., Wang Z., Cao X., Fu C. (2019). Enhanced piezoelectric response of (Ba,Ca)(Ti,Zr)O_3_ ceramics by super large grain size and construction of phase boundary. J. Alloy. Compd..

[75] Acosta M., Novak N., Rojas V., Patel S., Vaish R., Koruza J., Rossetti G.A., Rödel J. (2017). BaTiO_3_-based piezoelectrics: Fundamentals, current status, and perspectives. Appl. Phys. Rev..

[76] Waqar M., Wu H., Chen J., Yao K., Wang J. (2022). Evolution from lead-based to lead-free Piezoelectrics: Engineering of lattices, domains, boundaries, and defects leading to giant response. Adv. Mater..

[77] Sluka T., Tagantsev A.K., Damjanovic D., Gureev M., Setter N. (2012). Enhanced electromechanical response of ferroelectrics due to charged domain walls. Nat. Commun..

[78] Fu Q., Saiz E., Rahaman M.N., Tomsia A.P. (2013). Toward strong and tough glass and ceramic scaffolds for bone repair. Adv. Funct. Mater..

[79] Gerhardt L.C., Boccaccini A.R. (2010). Bioactive glass and glass-ceramic scaffolds for bone tissue engineering. Materials.

[80] Morgan E.F., Unnikrisnan G.U., Hussein A.I. (2018). Bone mechanical properties in healthy and diseased states. Annu. Rev. Biomed. Eng..

[81] Zhang H.-Y., Zeng Z.-Y., Zhao Y.-Q., Lu Q., Cheng Y. (2016). First-principles study of lattice dynamics, structural phase transition, and thermodynamic properties of barium titanate. Z. Für Naturforsch. A.

[82] Rho J.-Y., Tsui T.Y., Pharr G.M. (1997). Elastic properties of human cortical and trabecular lamellar bone measured by nanoindentation. Biomaterials.

[83] Wang X., Chen X., Hodgson P., Wen C. (2006). Elastic modulus and hardness of cortical and trabecular bovine bone measured by nanoindentation. Trans. Nonferrous Met. Soc. China.

[84] Behrens B.-A., Wirth C.J., Windhagen H., Nolte I., Meyer-Lindenberg A., Bouguecha A. (2008). Numerical investigations of stress shielding in total hip prostheses. Proc. Inst. Mech. Eng. Part H J. Eng. Med..

[85] Zhang M., Gregory T., Hansen U., Cheng C.-K. (2020). Effect of stress-shielding-induced bone resorption on glenoid loosening in reverse total shoulder arthroplasty. J. Orthop. Res..

[86] Liverani E., Rogati G., Pagani S., Brogini S., Fortunato A., Caravaggi P. (2021). Mechanical interaction between additive-manufactured metal lattice structures and bone in compression: Implications for stress shielding of orthopaedic implants. J. Mech. Behav. Biomed. Mater..

[87] Rana M., Chaudhuri A., Biswas J.K., Karim S.I., Datta P., Karmakar S.K., Roychowdhury A. (2021). Design of patient specific bone stiffness mimicking scaffold. Proc. Inst. Mech. Eng. Part H J. Eng. Med..

[88] Ribeiro C., Sencadas V., Correia D.M., Lanceros-Méndez S. (2015). Piezoelectric polymers as biomaterials for tissue engineering applications. Colloids Surf. B Biointerfaces.

[89] Zheng T., Yu Y., Pang Y., Zhang D., Wang Y., Zhao H., Zhang X., Leng H., Yang X., Cai Q. (2022). Improving bone regeneration with composites consisting of piezoelectric poly(l-lactide) and piezoelectric calcium/manganese co-doped barium titanate nanofibers. Compos. Part B Eng..

[90] Goonoo N., Bhaw-Luximon A. (2022). Piezoelectric polymeric scaffold materials as biomechanical cellular stimuli to enhance tissue regeneration. Mater. Today Commun..

[91] Yang C., Ji J., Lv Y., Li Z., Luo D. (2022). Application of piezoelectric material and devices in bone regeneration. Nanomaterials.

[92] Damaraju S.M., Wu S., Jaffe M., Arinzeh T.L. (2013). Structural changes in PVDF fibers due to electrospinning and its effect on biological function. Biomed. Mater..

[93] Teixeira L.N., Crippa G.E., Gimenes R., Zaghete M.A., de Oliveira P.T., Rosa A.L., Beloti M.M. (2011). Response of human alveolar bone-derived cells to a novel poly(vinylidene fluoride-trifluoroethylene)/barium titanate membrane. J. Mater. Sci. Mater. Med..

[94] Panda A.K., Sitaramgupta V.S.N., Pandya H.J., Basu B. (2022). Electrical stimulation waveform-dependent osteogenesis on PVDF/BaTiO_3_ composite using a customized and programmable cell stimulator. Biotechnol. Bioeng..

[95] Teixeira L.N., Crippa G.E., Trabuco A.C., Gimenes R., Zaghete M.A., Palioto D.B., de Oliveira P.T., Rosa A.L., Beloti M.M. (2010). In vitro biocompatibility of poly(vinylidene fluoride–trifluoroethylene)/barium titanate composite using cultures of human periodontal ligament fibroblasts and keratinocytes. Acta Biomater..

[96] Lopes H.B., Santos T.d.S., de Oliveira F.S., Freitas G.P., de Almeida A.L., Gimenes R., Rosa A.L., Beloti M.M. (2014). Poly(vinylidene-trifluoroethylene)/barium titanate composite for in vivo support of bone formation. J. Biomater. Appl..

[97] Gimenes R., Zaghete M.A., Bertolini M., Varela J.A., Coelho L.O., Silva N.F., Bar-Cohen Y. (2004). Composites PVDF-TrFE/BT used as bioactive membranes for enhancing bone regeneration. Proceedings of the Smart Structures and Materials 2004: Electroactive Polymer Actuators and Devices (EAPAD).

[98] dos Santos G.G., Malherbi M.S., de Souza N.S., César G.B., Tominaga T.T., Miyahara R.Y., de Mendonça P.d.S.B., Faria D.R., Rosso J.M., Freitas V.F. (2022). 4th generation biomaterials based on PVDF-hydroxyapatite composites produced by electrospinning: Processing and characterization. Polymers.

[99] Karimi S., Ghaee A., Barzin J. (2019). Preparation and characterization of a piezoelectric poly (vinylidene fluoride)/nanohydroxyapatite scaffold capable of naproxen delivery. Eur. Polym. J..

[100] Rodrigues P.J.G., Elias C.d.M.V., Viana B.C., de Hollanda L.M., Stocco T.D., de Vasconcellos L.M.R., Mello D.d.C.R., Santos F.E.P., Marciano F.R., Lobo A.O. (2020). Electrodeposition of bactericidal and bioactive nano-hydroxyapatite onto electrospun piezoelectric polyvinylidene fluoride scaffolds. J. Mater. Res..

[101] Malherbi M.S., Dias L.C., Lima M.S.Z., Ribeiro L.G., Freitas V.F., Bonadio T.G.M., Silva L.M., Souza G.B., Volnistem E.A., Rosso J.M. (2022). Electrically stimulated bioactivity in hydroxyapatite/β-tricalcium phosphate/polyvinylidene fluoride biocomposites. J. Mater. Res. Technol..

[102] Ribeiro C., Correia D.M., Rodrigues I., Guardão L., Guimarães S., Soares R., Lanceros-Méndez S. (2017). In vivo demonstration of the suitability of piezoelectric stimuli for bone reparation. Mater. Lett..

[103] Reis J., Frias C., Canto e Castro C., Botelho M.L., Marques A.T., Simões J.A.O., Capela e Silva F., Potes J. (2012). A new piezoelectric actuator induces bone formation in vivo: A preliminary study. J. Biomed. Biotechnol..

